# Pentylene glycol: An emerging cosmetic allergen?

**DOI:** 10.1111/cod.13963

**Published:** 2021-09-13

**Authors:** Monica Corazza, Cecilia Schenetti, Natale Schettini, Pierantonia Zedde, Alessandro Borghi

**Affiliations:** ^1^ Department of Medical Sciences, Section of Dermatology University of Ferrara Ferrara Italy

**Keywords:** 1,5‐pentanediol, allergic contact dermatitis, CAS no 5343‐92‐0, case report, cosmetic cream, glycols, pentylene glycol

## CASE REPORT

A 44‐year‐old female patient was referred for patch testing because of two episodes of severe face eczema. The patient reported that the dermatitis developed after the use of the anti‐aging cream Bionike Defence My Age Day Cream® (ICIM International, Milano, Italy) and was successfully treated with an oral corticosteroid.

**FIGURE 1:**
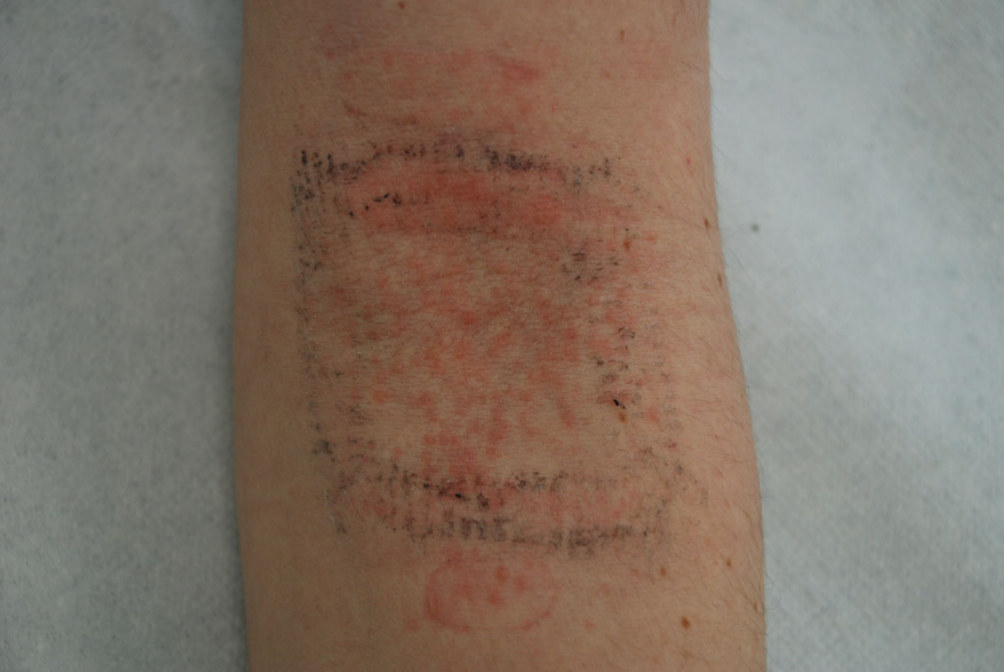
The positive repeated open application test (ROAT) performed with Bionike Defence My Age Cream® in the patientʼs antecubital fossa

Patch testing was performed with the SIDAPA (Società Italiana di Dermatologia Allergologica Professionale e Ambientale) baseline series and the integrative eyelids series (F.I.R.M.A., Florence, Italy). Patch‐test chambers (Van der Bend, Brielle, The Netherlands) were applied on the upper part of the patientʼs back. The readings on day (D) 2 and D3, according to the Italian guidelines,^1^ showed positive reactions to nickel sulfate (+++/+++) and cobalt chloride (+/++). The patient did not inform us of any additional patch test reactions beyond D3. A repeated open application test (ROAT) with Bionike cream in the antecubital fossa was performed; a positive reaction was observed within 3 days, confirming that the cream was the agent responsible for the patientʼs face eczema (Figure 1). Due to the ROAT strong reaction, the patient refused further patch test with the cream.

The label on the cream reported pentylene glycol (PTG) among the top ingredients. As we were not able to contact the manufacturer, we performed patch tests only with PTG 5% and 10% aq.; both the concentrations gave a positive reaction (+) at D3. PTG was obtained from Symrise (Holzminden, Germany) and tested at 5% and 10% concentrations according to previous studies.^2^, ^3^, ^4^, ^5^, ^6^, ^7^

We also tested propylene glycol (PG) 5% pet. and 30% aq., with no reaction.

Patch tests with PTG and PG at the same concentrations were performed in 15 healthy subjects with no reactions.

## DISCUSSION

Pentylene glycol (1,5‐pentanediol; 1,2‐dihydroxy pentane; CAS no. 5343‐92‐0) is a preservative, solvent, and humectant that might be used increasingly in cosmetic products. It is considered to be both a weak irritant and a weak allergen.

Few cases of sensitization to PTG have been described in the literature^2^, ^3^, ^4^, ^5^, ^6^, ^7^ (Table 1). Most of the cases involved the facial region, and a previous dermatitis was present.

**TABLE 1:** Previous reported cases of allergic contact dermatitis due to pentylene glycol

Authors	Gender/age	Site of contact dermatitis	Clinical aspects	Preexisting dermatosis	Product	Patch test with product (“as is”) and/or ROAT	Patch test with pentylene glycol	Patch test with propylene glycol	Concomitant allergens
Gallo et al^3^	F/90	Eyelids and periorbital region	Severe eczema	No	Toleriane Ultra Yeux® (eye cream)	D2+/D3+ ROAT pos D2	5% water/alcohol D2+/D4+	Negative	Benzyl alcohol + D3
Foti et al^4^	M/62	Face	Worsening of seborrheic dermatitis	Seborrheic dermatits	Sebclair face cream® (face cream)	D2++/D3++	2% pet. D2++/D3++ 5% pet. D2++/D3++ 5% aq. D2++/D3++	5% pet. +	Corticosteroids
Mortz et al^5^	F/68	Face	Recurrent and spreading dermatitis	No	Decubal face cream® Decubal eyes cream®	D3++	5% aq. D3+ 0.5% aq. D3+	Negative	Ethylhexylglycerin
Kerre et al^6^	F/56	Face and eyelids	Dermatitis and swelling of the eyelids	No	Toleriane riche cream®, (face cream) L. Widmer body lotion®	D2++	5% aq. Neg ROAT pos D3	5% aq. +	No
Amado et al^7^	F/44	Face and body	Scaling erythematous dermatitis	Atopic dermatitis	MimyX cream® (body emulsion)	D2++/D3++ ROAT D7++	5% pet. D2+++/D3+++ 10% pet. D2+++/D3+++	Not performed	Fragrance mix I, paraphenylenediamine, neomycin, tea tree oil
Gallo et al^2^	M/39	Skin folds	Exudative and itchy dermatitis	Scaling erythematous dermatitis	Resvelife cream® (body cream)	D2+/D3++	5% aq. D2++ /D3++ 10% aq. D2++/D3++ 0.5% aq. D2+/D3++	Negative	Resveratrol

Due to the similar chemical structure between PTG and PG, differing only in a longer carbon chain and the position of alcohol groups in PTG, a cross‐reaction could be expected. In our patient no cross‐reactions were observed, confirming what has been observed in the literature.

Further studies are strongly needed define the real allergenic potential of this molecule, which is used frequently in cosmetics that are formulated for sensitive and atopic skin. It is also desirable to determine the correct concentration and the vehicle for PTG to be used in patch tests. Finally, the occurrence of cross‐reactivity between different glycols should be deeply investigated, perhaps using higher patch‐test concentrations and later readings.

## CONFLICT OF INTERESTS

The authors declare no funding and no conflicts of interest related directly to the work being submitted.

## AUTHOR CONTRIBUTIONS

**Monica Corazza:** Conceptualization (lead); data curation (lead); formal analysis (lead); investigation (lead); methodology (lead); writing – review and editing (lead). **Cecilia Schenetti:** Conceptualization (equal); data curation (equal); formal analysis (equal); investigation (equal); writing – original draft (lead); writing – review and editing (supporting). **Natale Schettini:** Data curation (supporting); investigation (supporting). **Pierantonia Zedde:** Data curation (supporting); investigation (supporting). **Alessandro Borghi:** Formal analysis (equal); investigation (equal); supervision (lead); validation (equal); writing – review and editing (supporting).
